# Dietary Carotenoid Intake and Sleep: A Narrative Review of Evidence from Human and In Vivo Animal Studies

**DOI:** 10.3390/nu18172833

**Published:** 2026-08-28

**Authors:** Josephine Dudzik, Lucy Weaver, Sanja Jelic, Marie-Pierre St-Onge

**Affiliations:** 1 Center of Excellence for Sleep & Circadian Research, Columbia University Medical Center, New York, NY 10032, USA; jmd2336@cumc.columbia.edu; 2 Institute of Human Nutrition, Vagelos College of Physicians and Surgeons, Columbia University Irving Medical Center, New York, NY 10032, USA; 3 Division of Pulmonary, Allergy, and Critical Care Medicine, Columbia University Medical Center, New York, NY 10032, USA; 4 Division of General Medicine, Columbia University Medical Center, New York, NY 10032, USA

**Keywords:** carotenoids, sleep, sleep health, diet, phytochemicals, antioxidants, inflammation

## Abstract

**Background/Objectives:** Carotenoids are dietary bioactive compounds found primarily in red, yellow, and orange fruits and vegetables that possess antioxidant and anti-inflammatory properties. These properties are increasingly known to be relevant for sleep health. Hence, this narrative review aims to summarize current evidence on the relation between carotenoids and sleep. **Methods:** PubMed and Scopus were searched for observational and intervention studies in adults and in vivo preclinical studies examining carotenoid exposure in relation to sleep-related outcomes. Eligible studies were screened using predefined criteria, and findings were synthesized narratively. **Results:** Of the 1042 studies screened, 29 met the inclusion criteria, including 14 observational, 11 intervention, and 4 preclinical studies. Across observational studies, higher dietary intake or serum carotenoid concentrations were generally associated with more favorable sleep outcomes, including sleep quality, duration, and sleep-related symptoms, although heterogeneous sleep-outcome definitions limited cross-study comparison. Nine intervention studies (81.8%) reported improvement in at least one sleep-related outcome following carotenoid supplementation. However, improvement was not consistently significant compared with placebo. Preclinical studies provided preliminary evidence that selected carotenoids improved sleep–wake parameters, including dose-dependent increases in total sleep and non-REM sleep duration, as well as reductions in wakefulness. **Conclusions:** Emerging evidence suggests that carotenoids may contribute to sleep health. However, current evidence remains limited and heterogeneous. Additional research is needed to determine the effects of specific carotenoids, optimal doses, and underlying biological mechanisms to better inform evidence-based clinical practice recommendations.

## 1. Introduction

Sleep is a fundamental determinant of overall health and well-being, and inadequate sleep is increasingly recognized as a risk factor for a wide range of adverse cardiometabolic outcomes, such as obesity, type 2 diabetes, and cardiovascular disease [1,2,3,4]. Sleep duration is now recognized as a component of the American Heart Association’s Life’s Essential 8 [5], a measure of cardiovascular health that predicts cardiovascular disease events and mortality [6]. Yet, emerging evidence indicates that multidimensional sleep health, which includes sleep duration, timing, regularity, daytime functioning, sleep architecture (stages), and absence of sleep disorders, also impacts cardiometabolic disease risk and related risk factors [7,8]. Given the global burden of insufficient sleep [9], identifying strategies to improve sleep health has the potential to help mitigate the high prevalence of chronic disease.

Among the modifiable lifestyle factors that influence sleep regulation, diet has emerged as an important contributor [10]. Adherence to a plant-based dietary pattern, such as a Mediterranean diet, has been associated with improved multidimensional sleep health [11,12,13]. One proposed mechanism underlying this association involves the anti-inflammatory properties of a plant-based diet [14], which may help address the systemic inflammation commonly observed both in inadequate sleep and cardiometabolic disease [15,16]. Supporting this, pro-inflammatory diets, often quantified using the energy-adjusted dietary Inflammatory Index (e-DII), are linked to elevated circulating inflammatory biomarkers [17], poorer sleep health [17,18,19], and an increased risk of cardiometabolic disease [17,19].

Plant-derived dietary bioactive compounds, commonly referred to as phytochemicals, are key constituents of plant-based diets and are thought to contribute to their health benefits through anti-inflammatory and antioxidant activities [20]. Higher dietary phytochemical intake, derived primarily from fruits, vegetables, whole grains, nuts, and legumes, has also been associated with improved multidimensional sleep health [21,22,23]. Among these compounds are carotenoids, which are lipid-soluble, naturally occurring red, yellow, and orange pigments found abundantly in fruits and vegetables. Carotenoids comprise two major classes, carotenes and xanthophylls, along with several subclasses, such as apocarotenoids and norcarotenoids [24]. The predominant dietary carotenoids include α-carotene, β-carotene, β-cryptoxanthin, lutein and zeaxanthin, and lycopene, which are found in a variety of red, yellow, and orange fruits and vegetables, as well as leafy green vegetables.

Carotenoids positively influence health through their ability to modulate inflammatory and oxidative stress pathways [25,26]. Although their beneficial effect on cardiometabolic risk is well established, no known review has comprehensively synthesized current evidence on the relation between carotenoids and sleep health. Addressing this knowledge gap may improve our understanding of the mechanisms linking diet, sleep, and cardiometabolic disease risk. Therefore, the primary objective of this narrative review is to synthesize current evidence on the relation between dietary carotenoid intake and sleep in humans and in vivo animal models. We also examine the association between serum carotenoid concentrations and sleep and discuss potential mechanisms underlying these relations.

## 2. Methods

We conducted a structured literature search using PubMed and Scopus electronic databases for potentially eligible studies published before July 2026. Search queries included at least one search term relevant to the research objective, including “carotenoids”, “alpha-carotene”, “beta-carotene”, “lycopene”, “lutein”, “zeaxanthin”, and “astaxanthin”, as well as their associated medical subject headings (MeSH). These terms were cross-referenced with those related to predefined outcomes of interest, including “sleep”, “sleep quality”, “sleep duration”, “sleep disorders”, “sleep-disordered breathing”, and “sleep architecture” using advanced search building tools. Reference lists of included studies and relevant literature reviews were manually screened to identify additional articles relevant for inclusion. The search strategy is detailed in the Supplementary Materials.

Studies were eligible for inclusion if they were original research involving clinical trials, preclinical trials, or observational designs and included free-living adults (≥18 years) or in vivo animal models. Exposures of interest included dietary carotenoid intake, measured as either carotenoid source totals or individual carotenoids, carotenoid-containing dietary supplements, or serum carotenoid concentrations. Studies were excluded if they investigated multiple bioactive compounds simultaneously, evaluated mixed diets or whole foods, utilized dietary supplements containing mixed nutrients, or otherwise did not permit assessment of carotenoid-specific effects. Outcomes of interest were broadly related to sleep, such as sleep quality, duration, disorders, sleep-disordered breathing, and sleep architecture, and could be measured subjectively by self-report/questionnaire or objectively. Gray literature, conference abstracts, and studies not published in peer-reviewed journals were excluded, as were those not published or available in English.

Identified studies were screened and reviewed using Covidence, a web-based collaboration software platform (Veritas Health Innovation, Melbourne, Australia). Title and abstract screening and full-text review were conducted by two independent reviewers (J.D. and L.W.). In case of conflict, authors met to reach consensus among all members of the research team. Data were extracted independently using data extraction tables developed specifically for this review and organized by population (human subjects or in vivo animal models) and study design (intervention or observational). A second study team member conducted serial checks of extracted data to ensure accuracy. Although conducted as a narrative review with a structured search strategy, literature identification and study selection were guided by the Preferred Reporting Items for Systematic Reviews and Meta-Analyses (PRISMA) 2020 statement to enhance the transparency and reproducibility of the review methods. Due to substantial heterogeneity in study designs (observational, clinical, and preclinical), carotenoid assessment methods (e.g., serum concentrations and dietary intake), sleep outcomes and their assessment (e.g., outcome definitions and measurement methods), population characteristics (e.g., health status, sex, and age range), and intervention characteristics (e.g., carotenoid type, intervention duration, and comparator group), findings were synthesized narratively and meta-analyses were not conducted.

## 3. Results

**Figure 1:**
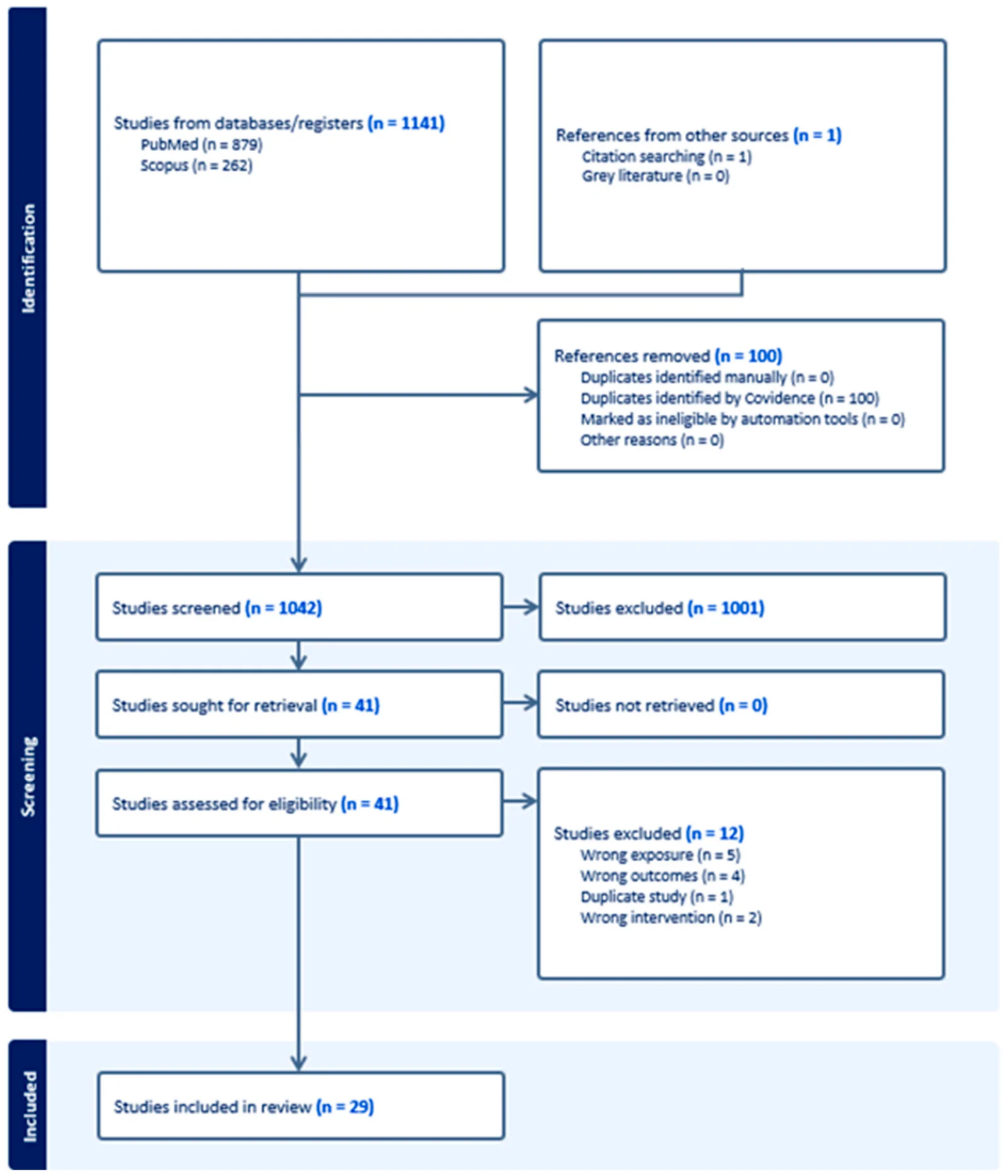
Preferred reporting items for systematic reviews and meta-analyses flow diagram.

A total of 1142 articles were identified from the initial search, and after the removal of 100 duplicates, 1042 articles were screened by title and abstract, of which 1001 were excluded (Figure 1). Of the 41 articles screened during the full-text screening phase, 29 met the eligibility criteria and were included in this review. Studies were classified as human observational (*n* = 14) [27,28,29,30,31,32,33,34,35,36,37,38,39,40], human intervention (*n* = 11) [41,42,43,44,45,46,47,48,49,50,51], or preclinical (*n* = 4) [52,53,54,55].

### 3.1. Evidence from Human Observational Studies

Study characteristics of included human observational studies (*n* = 14; sample sizes ranging from 18 to 24,234 participants) are described in Table 1. Of these, eight studies examined the association between dietary carotenoid intake and sleep parameters [28,29,30,32,33,34,35,39], five studies focused on serum carotenoid concentrations [31,36,37,38,40], and one study examined both [27]. In total, nine articles reported data from the National Health and Nutrition Examination Survey (NHANES) collected across annual survey cycles between 2005 and 2018, with each survey cycle including a newly selected participant sample [27,30,32,33,34,35,36,37,40]. All studies utilized a cross-sectional observational design with the exception of one cross-sectional case–control study [31].

#### 3.1.1. Dietary Carotenoid Intake and Sleep

Sleep quality was evaluated in three studies, all of which utilized the Pittsburgh Sleep Quality Index (PSQI) [28,29,39]. Two studies measured dietary β-carotene intake [29,39], whereas one examined total dietary carotenoid intake [28], calculated as the sum of intakes of the five major carotenoids (α-carotene, β-carotene, β-cryptoxanthin, lutein + zeaxanthin, and lycopene). Both studies evaluating the association between β-carotene intake and sleep quality reported higher β-carotene intakes among participants with better sleep quality, including adults aged 20–64 years (PSQI scores ≤ 5 versus >5) [29] and young Japanese male university students (PSQI scores ≤ 3 versus ≥6) [39]. Similarly, among university students, Boozari et al. found that higher total dietary carotenoid intake was associated with lower odds of poor sleep quality [28]. Compared with participants in the lowest intake quartile (Q1), those in Q3 and Q4 had 71% and 81% lower odds of poor sleep quality (PSQI score ≥ 6), respectively [28].

Four studies analyzed the association between dietary carotenoid intake and sleep duration, three of which measured sleep duration from NHANES questionnaire data [27,33,35] and one from the PSQI [29]. Two studies evaluated total carotenoid intake [27,33], three assessed all five major carotenoids individually [27,33,35], and one evaluated β-carotene intake alone [29]. Compared with adults with short (<7 h) or long (>8–9 h) sleep duration, adults with optimal sleep duration (7–8 h) had higher carotenoid intake [27,33], suggesting a potential inverse U-shaped association between total carotenoid intake and sleep duration that warrants further investigation [33]. Deng et al. further reported that higher combined intakes of all five major dietary carotenoids were associated with lower odds of both short and long sleep duration relative to optimal sleep duration [33]. In this analytical model, β-cryptoxanthin and lutein + zeaxanthin showed the strongest individual associations with lower odds of short sleep duration, whereas α-carotene, β-carotene, and β-cryptoxanthin showed the strongest individual associations with lower odds of long sleep duration [33].

When examined individually, lower dietary intakes of α-carotene, β-carotene, β-cryptoxanthin, lutein + zeaxanthin, and lycopene were associated with higher odds of short sleep duration compared with optimal sleep duration in one study [33]. In studies that compared dietary intakes across sleep-duration categories, adults with short or very short sleep duration had lower intakes of α-carotene, β-carotene, and lutein + zeaxanthin than those with optimal sleep duration [27], while adults with very short sleep duration also had lower lycopene intake than those with optimal sleep duration [35].

Evidence for long sleep duration was less consistent. Lower intakes of each of the five major carotenoids were independently associated with higher odds of long sleep duration compared with optimal sleep duration in one study [33], whereas in another study, adults with long sleep duration had lower lutein + zeaxanthin intake than those with optimal sleep duration [27]. In contrast, Çakir et al. found no significant differences in dietary β-carotene intake across short (<6 h), optimal (6–8 h), and long (>8 h) sleep duration categories [29]. Notably, the thresholds used to define short and long sleep duration varied across studies, further limiting direct comparisons of these findings.

Three additional studies evaluated other sleep outcomes, with findings suggesting potential associations that require confirmation [30,32,34]. In individual studies, participants in Q3 of dietary lycopene intake had lower odds of reporting that they had ever told a health professional about trouble sleeping compared with those in the lowest quartile (Q1) [30], while a 100% higher dietary lycopene intake was associated with 2% lower odds of difficulty maintaining sleep [34]. In another study, higher dietary lycopene intake was also associated with higher sleep regularity index (SRI) [32]. Furthermore, a 100% higher α-carotene intake was associated with 4% lower odds of difficulty falling asleep [34]. Given the limited evidence for individual sleep outcomes and variability in their assessment, further studies are needed to establish the reproducibility of these associations.

#### 3.1.2. Serum Carotenoid Concentration and Sleep

Four studies investigated associations between serum carotenoid concentrations and sleep duration using a range of carotenoid biomarkers and sleep duration measures [27,36,37,38]. Lower total serum carotenoid concentrations were generally observed among adults with short sleep duration compared with adults sleeping 7–8 h/night [27,36,38]. Lower serum concentrations of several individual carotenoids, including β-carotene [27,38], lycopene [38], α-carotene [27], and β-cryptoxanthin [27], were also associated with increased risk of short sleep duration. Similarly, lower concentrations of four of the five major carotenoids (excluding lycopene) were associated with increased risk of abnormal sleep duration [37]. In contrast to these inverse associations, Beydoun et al. reported that, after adjustment for multiple comparisons, higher total serum carotenoid concentrations were associated with a 15% greater risk of short sleep (5–6 h) relative to normal sleep duration (7–8 h) [27].

Three studies examined additional sleep outcomes [27,31,37]. In a case–control study of adults with obstructive sleep apnea (OSA), plasma concentrations of all-trans and 9-cis β-carotene were 50% and 35% lower, respectively, than in age- and sex-matched healthy controls [31]. Higher serum carotenoid concentrations were associated with lower odds of reporting trouble sleeping, frequent snoring, and witnessed breathing interruptions during sleep in one study [37], with reduced sleepiness and sleep disturbance in another [27]. However, findings within the latter study were mixed, as Beydoun et al. also reported that higher serum total carotenoids, β-carotene, and β-cryptoxanthin were associated with greater self-reported daytime sleepiness, while higher serum lutein + zeaxanthin were associated with greater daytime dysfunction among adults in NHANES 2005–2006 [27]. These divergent findings preclude firm conclusions regarding the relation between serum carotenoid concentrations and these sleep outcomes, warranting confirmation in additional studies.

Only one study examined serum carotenoid concentrations in relation to sleep quality; no significant differences were noted across sleep quality categories [40].

### 3.2. Evidence from Human Intervention Studies

Table 2 describes study and intervention characteristics of the 11 human intervention studies discussed in this review. Study designs were parallel-group randomized controlled trials (RCT; *n* = 7) [43,44,45,47,48,49,51], crossover RCTs including one pilot study (*n* = 3) [41,46,50], and a nonrandomized pilot study (*n* = 1) [42], with sample sizes ranging from 20 to 121 adults. Interventions included supplementation with xanthophyll-class carotenoids such as lutein + zeaxanthin (*n* = 3) [41,47,48], β-cryptoxanthin (*n* = 1) [49], astaxanthin (*n* = 1) [43], and fucoxanthin (*n* = 1) [51]; apocarotenoids such as crocin (*n* = 2) [44,45] and crocetin (*n* = 2) [46,50]; and lycopene (*n* = 1), a carotene-class carotenoid [42].

#### 3.2.1. Xanthophyll-Class Intervention Studies

Two RCTs evaluated supplementation with 10 mg/day lutein plus 2 mg/day zeaxanthin in adults with prolonged screen time (≥6 h/day) [41,47]. One was a 6-month placebo-controlled RCT in which participants received one dose daily [47]; the other was an 8-month crossover RCT in which participants received two doses daily [41]. In this latter study, participants in group A received the intervention throughout the entire study, group B received the intervention for 4 months followed by placebo for 4 months, and group C received placebo for 4 months followed by the intervention for 4 months (the reverse of group B), with a 15-day washout period between the two 4-month treatment periods [41]. A third placebo-controlled RCT in healthy college-age adults with ≥6 h/day of near-field screen exposure evaluated a dietary supplement containing 24 mg/day lutein, zeaxanthin, and meso-zeaxanthin in an 83:10:7 ratio for 6 months [48].

Across the three lutein- and zeaxanthin-containing interventions, several self-reported sleep outcomes generally improved from baseline or relative to placebo, although evidence of a supplementation-specific effect was inconsistent. Sleep quality was assessed in two studies using the Quality of Sleep Questionnaire [41] and the PSQI [48]. In the 8-month crossover dual-supplement RCT, participants receiving lutein and zeaxanthin during the initial 4-month intervention experienced significant improvements from baseline in sleep duration, nighttime awakenings, and feelings of relaxation upon waking [41]. In contrast, participants receiving placebo during the first 4 months showed no significant changes from baseline in any sleep quality domain [41]. Following the crossover, participants who switched from placebo to lutein and zeaxanthin experienced significant improvements in sleep duration, nighttime awakenings, feelings of relaxation upon waking, and how their eyes felt upon waking compared with both baseline and the end of the placebo period [41]. Among participants who switched from lutein and zeaxanthin to placebo, improvements achieved during active supplementation were largely maintained throughout the placebo period [41]. Participants who continued lutein and zeaxanthin supplementation for the full 8 months also maintained their improvements through the end of the study [41].

Similarly, in a 6-month supplementation intervention with lutein, zeaxanthin, and meso-zeaxanthin, sleep quality improved significantly relative to placebo after both 3 and 6 months [48]. This study also reported a decrease from baseline in the mean number of self-reported undesirable sleep symptoms in the supplementation group, with reductions that were significant relative to placebo at both 3 and 6 months [48]. In the third 6-month lutein and zeaxanthin intervention study, sleep-related impairment significantly improved from baseline to endpoint in both the intervention and placebo groups, with no significant between-group differences [47].

Findings from studies evaluating other xanthophyll carotenoids were mixed, with some within-group improvements in sleep-related outcomes following astaxanthin [43] and fucoxanthin supplementation [51], but no significant changes following β-cryptoxanthin supplementation [49]. Astaxanthin supplementation at a dose of 12 mg/day for 8 weeks was associated with significant within-group improvements from baseline in self-reported sleepiness upon awakening after 4 and 8 weeks and feelings of being refreshed after 4 weeks; however, these changes did not differ significantly from placebo [43]. Yet, in a subgroup of participants with high Depression–Dejection scores, sleepiness upon waking improved significantly with astaxanthin relative to placebo. Among older adults with self-perceived cognitive and memory decline, fucoxanthin supplementation at 8.8 mg/day for 12 weeks was associated with significant improvements from baseline in ease of awakening and time to feeling alert after 4 weeks, although no significant overall group × time effects were observed [51]. Finally, among healthy young adult women, β-cryptoxanthin supplementation at 3 or 6 mg/day for 8 weeks resulted in no significant within- or between-group changes in self-reported sleep quality or objectively measured sleep duration [49].

Collectively, although some xanthophyll-class carotenoid interventions were associated with improvements in sleep-related outcomes, inconsistent between-group effects and the limited number of trials for individual carotenoids limit our ability to draw definitive conclusions regarding supplementation-specific effects.

#### 3.2.2. Apocarotenoid-Class Intervention Studies

Four studies evaluated supplementation with the apocarotenoids crocin, safranal, and crocetin, a class of carotenoids produced through the oxidative cleavage of the xanthophyll carotenoid zeaxanthin [44,45,46,50]. Two studies investigated crocin supplementation, with sleep quality measured by the PSQI as the primary outcome [44,45]. Among healthy adults with self-reported low mood but no diagnosed depression, there were no significant improvements in sleep quality from baseline following 4 weeks of supplementation with either 22 or 28 mg/day of standardized saffron extract containing >3.5% Lepticrosalides^©^, a combination of saffron bioactive compounds that included crocin and safranal [44]. In contrast, supplementation with 30 mg/day of purified crocin for 8 weeks significantly improved sleep quality compared with placebo in an exclusively male cohort with substance dependence undergoing methadone maintenance treatment [45].

The remaining two studies evaluated 7.5 mg/day crocetin supplementation for 2 weeks using crossover RCT designs [46,50]. Both assessed subjective and objective sleep outcomes. In healthy adult men with mild sleep complaints (PSQI ≥6), crocetin supplementation significantly reduced the number of wake episodes compared with placebo, whereas the observed improvement in self-reported sleep quality from baseline was not statistically significant [46]. In the other RCT, crocetin supplementation significantly increased electroencephalography (EEG)-derived delta power measured during non-REM sleep in the first sleep cycle, compared with placebo in healthy men and postmenopausal women with mild sleep complaints [50]. In addition, participants reported significantly less sleepiness on waking and greater feelings of refreshment following crocetin supplementation than after placebo [50].

#### 3.2.3. Carotene-Class Intervention Studies

In an exclusively male cohort aged ≥ 50 years with and without benign prostatic hyperplasia, participants received 8 mg/day of lycopene in a lycopene-enriched extra-virgin olive oil product for 30 days to evaluate effects on sleep quality as measured by actigraphy [42]. In men both with and without benign prostatic hyperplasia, there was a significant decrease in the % of nighttime activity at postintervention compared to baseline; however, nighttime sleep duration and sleep depth did not significantly change in either group [42].

Across intervention studies, improvements were observed for some sleep-related outcomes relative to baseline or placebo; however, findings were not consistent across outcomes or carotenoid interventions. Differences in carotenoid formulation and dose, intervention duration, study population, and sleep outcomes and assessment methods limit comparisons across studies. Given the small number of trials evaluating each carotenoid and the variability in observed effects, additional RCTs are needed to determine whether these findings are reproducible.

### 3.3. Evidence from Preclinical Studies

Table 3 describes the characteristics of the four preclinical studies included in this review. Three studies utilized male mice [52,53,55], while one utilized male and female *Drosophila melanogaster* [54]. Two studies examined apocarotenoids, including crocin, crocetin, and safranal [52,53], and two examined the xanthophyll-class carotenoids fucoxanthin and astaxanthin [54,55]. Sleep-related outcomes included total sleep time, daytime and nighttime sleep or rest, wakefulness, non-rapid eye movement (non-REM) and REM sleep, wake-bout frequency and duration, and transitions between sleep and wake states.

#### 3.3.1. Apocarotenoid-Class Preclinical Studies

Among the apocarotenoids, safranal was tested in one mouse study [52], while crocin and/or crocetin were examined in two mouse studies that produced contrasting findings [52,53]. In a pentobarbital-induced sleep model, intraperitoneal administration of safranal increased sleep duration in a dose-dependent manner at doses of 0.05, 0.15, and 0.35 mL/kg compared with the corresponding vehicle control, while crocin, at doses of 50, 200, and 600 mg/kg, did not significantly affect sleep duration relative to its vehicle control [52]. In contrast, an EEG-based assessment of sleep–wake states demonstrated dose-dependent increases in non-REM sleep and decreases in wakefulness relative to its vehicle control following intraperitoneal crocin administration [53]. Compared with vehicle controls, crocin at doses of 30 and 100 mg/kg increased total non-REM sleep by 60% and 170%, respectively, while reducing wakefulness by 20% and 50%, with no significant change in REM sleep [53]. Compared with vehicle-treated controls, at the 100 mg/kg dose, crocin also increased the number of non-REM sleep and wake bouts, shortened the mean wake-episode duration by 83%, and increased transitions from wakefulness to non-REM sleep [53]. Crocetin, administered at 100 mg/kg, similarly increased total non-REM sleep by 60% and reduced wakefulness by 25%, without affecting REM sleep, compared with vehicle control [53].

#### 3.3.2. Xanthophyll-Class Preclinical Studies

Among the xanthophyll-class carotenoids, fucoxanthin and astaxanthin were evaluated in one preclinical study each [54,55]. Fucoxanthin was administered to male and female *Drosophila melanogaster* at a concentration of 1 μM in yeast paste beginning on the first day of adult life, and sleep/rest was behaviorally defined as achieving at least five consecutive minutes of inactivity [54]. Fucoxanthin increased nighttime sleep/rest in males at one, four, and six weeks of age and in females at six weeks compared with untreated control flies [54]. During the daytime, fucoxanthin reduced sleep time in 1-week-old flies of both sexes but increased sleep in 4-week-old males and 6-week-old females, indicating that the effects of fucoxanthin on sleep/rest varied by age and sex in this model [54].

Astaxanthin was evaluated in a female mouse model of reserpine-induced fibromyalgia [55]. Following 10 days of intragastric administration, astaxanthin at 100 mg/kg/day significantly reduced wake duration and the number of wake bouts compared with untreated fibromyalgia control mice [55]. Astaxanthin also significantly increased non-REM sleep duration compared with untreated fibromyalgia controls without affecting REM sleep duration [55].

Collectively, these preclinical findings provide preliminary evidence that certain carotenoids may influence sleep–wake parameters under experimental conditions. However, the small number of studies, differences in species and experimental models, and contrasting findings limit their generalizability and relevance to human sleep health.

## 4. Dietary Sources and Biological Mechanisms of Carotenoids in Sleep Health

### 4.1. Classification, Dietary Sources, and Intake of Carotenoids

**Figure 2:**
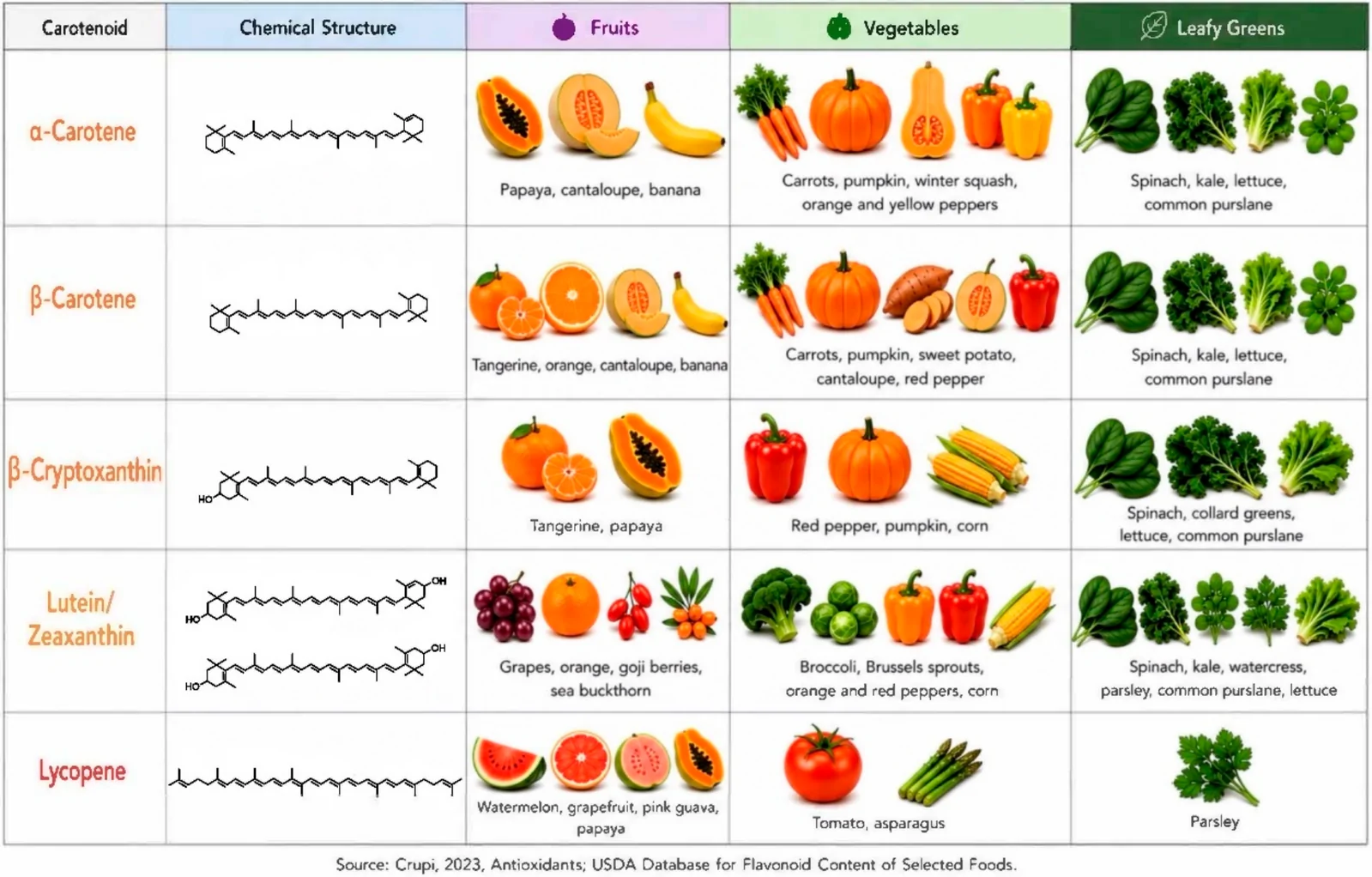
Food sources of major carotenoids [56].

Because carotenoids cannot be synthesized by the human body, they must be obtained through the diet, primarily from fruits and vegetables. Many of the carotenoids examined in this review are found in commonly consumed fruits and vegetables (Figure 2). Among the carotene class of carotenoids (i.e., hydrocarbons containing only carbon and hydrogen) are lycopene, a non-provitamin A carotenoid found in red fruits and vegetables, and the provitamin A carotenoids α-carotene and β-carotene, found in orange, yellow, and leafy green fruits and vegetables [56]. Major dietary xanthophyll-class carotenoids (i.e., oxygenated hydrocarbons) include β-cryptoxanthin, another provitamin A carotenoid found primarily in red and orange fruits and vegetables, and non-provitamin A carotenoids lutein and zeaxanthin, which are abundant in green leafy vegetables [56]. In contrast, other carotenoids are derived from less commonly consumed sources, including astaxanthin and fucoxanthin from marine algae, and the apocarotenoids crocin, crocetin, and safranal from saffron.

While consuming a varied plant-based diet can provide a range of carotenoids with the potential to support sleep health, epidemiological evidence indicates that carotenoid intake among U.S. adults has declined over the past decade from ~10,000 mcg/day in 2009–2010 to ~9690 mcg/day in 2017–2018 (*p*-trend = 0.097) [57]. Within the same time frame, the rate of vitamin A adequacy has decreased from 27.8% to 25.5% (*p*-trend <0.001), due in part to decreased average intakes of α-carotene [57]. Similar reductions in lycopene intake have likely been driven by significant decreases in tomato and vegetable juice intake over the past decade, although tomato products remain the major food source of carotenoids among U.S. adults [57]. Furthermore, trends in dietary carotenoid intake differ by sociodemographic characteristics such as sex, body mass index (BMI), household income, and physical activity, highlighting the need for equitable evidence-based public health recommendations that encourage consumption of carotenoid-rich foods.

### 4.2. Absorption, Metabolism, and Biological Fate of Carotenoids

As carotenoids are both lipophilic and highly hydrophobic compounds, their absorption from the food matrix requires solubilization in mixed micelles containing lipids and bile components after dispersion in the gastrointestinal tract [26,58]. This process, which increases in the presence of dietary fats [59], is a major determinant of carotenoid uptake by the intestinal epithelial cells and their ultimate bioavailability [58]. In the intestinal enterocytes, carotenoids are absorbed either by passive diffusion or by class B type 1 (SR-B1), Niemann–Pick C1-Like 1 (NPC1L1), and CD36 receptor-assisted transport [58,59].

In the enterocyte, carotenoid absorption is dependent on their chemical structure, though an estimated 40% of ingested carotenoids are not metabolized [60]. Provitamin A carotenoids, including α-carotene, β-carotene, and β-cryptoxanthin, can undergo enzymatic cleavage to retinal by the enzyme BCO1, which can be subsequently converted to other biologically active retinoids [61]. Carotenoids can also be converted into apocarotenoids by mitochondrial BCO2 [61]. Intact carotenoids are incorporated into nascent chylomicrons [59]. Once in circulation, intact carotenoids are transported and distributed to various peripheral tissues, primarily hepatic and adipose tissue, by high-density (HDL), low-density (LDL), and very low-density (VLDL) lipoproteins, though tissue distribution varies by carotenoid type [26,59].

Carotenoid bioavailability and subsequent tissue distribution are influenced by various dietary and internal factors, including carotenoid formulation, food matrix, degree of food processing, interactions with other dietary components, and inter-individual differences in absorptive and metabolic capacity [61,62]. Consequently, dietary carotenoid intake may not accurately reflect circulating or tissue carotenoid concentrations and differences in absorption, metabolism, and distribution should be considered when interpreting studies that use a single carotenoid exposure.

### 4.3. Potential Antioxidant Mechanisms of Action of Carotenoids on Sleep

The antioxidant properties of carotenoids may represent one mechanism underlying their beneficial effects on sleep regulation. However, conclusive evidence demonstrating that carotenoid-induced changes in antioxidant pathways mediate improvements in sleep in humans remains insufficient; therefore, the currently hypothesized mechanisms should be considered biologically plausible rather than established causal pathways. Extended sleep deprivation and chronic circadian disruption can disturb redox homeostasis, promoting oxidative stress and cellular damage [63,64]. For example, an in vivo study demonstrated that 72 h of sleep deprivation increased hepatic malondialdehyde and nitrite concentrations while reducing catalase activity and glutathione concentrations in mice compared with non-sleep-deprived controls [63]. Carotenoids may help counter these changes through both direct and indirect antioxidant actions [25]. The conjugated double-bond structure of carotenoids enables them to quench singlet oxygen and peroxyl radicals, thereby limiting oxidative damage.

Beyond direct radical scavenging, carotenoids may enhance endogenous antioxidant defenses through activation of the KEAP1-NRF2 signaling pathway. Upon activation and nuclear translocation, NRF2 induces the expression of genes involved in antioxidant and cytoprotective responses [25]. Evidence from in vitro studies supports this mechanism. In primary cortical neurons exposed to injury, fucoxanthin reduced intracellular reactive oxygen species (ROS), promoted nuclear translocation of NRF2, and increased expression of the downstream antioxidant and cytoprotective enzymes HO-1 and NQO1 [65]. Similarly, in MPP+-exposed PC12 neuronal cells, astaxanthin reduced intracellular ROS, increased NRF2 and HO-1 expression, and suppressed expression of the ROS-generating enzyme NOX2 [66]. Collectively, these findings suggest that carotenoids influence oxidative balance not only through direct radical scavenging but also by activating endogenous antioxidant defense pathways.

Evidence from animal models further supports these antioxidant mechanisms. In a circadian-disruption model, astaxanthin supplementation significantly increased serum catalase, glutathione peroxidase, and superoxide dismutase activities while reducing serum malondialdehyde concentrations in mice exposed to D-galactose and chronic jet lag [64]. Astaxanthin also significantly increased hepatic expression of *Gpx1*, *Sod1*, and *Sod2* and renal expression of *Sod1* and *Sod2*, further suggesting activation of endogenous antioxidant defense pathways [64]. Among the preclinical studies included in this review, fucoxanthin similarly increased *Sod3* mRNA expression in young male *Drosophila melanogaster*, although it did not improve resistance to paraquat-induced oxidative stress [54]. Consistent with these findings, astaxanthin pretreatment reduced ROS and malondialdehyde formation while preserving glutathione peroxidase and catalase activities in nerve growth factor-differentiated PC12 cells exposed to hydrogen peroxide or MPP+-induced oxidative stress [67].

Human observational evidence also provides indirect support for this proposed mechanism. In an analysis of NHANES 2007–2018 data, higher scores on a composite dietary antioxidant index that included carotenoids were associated with lower odds of poor sleep after adjustment for potential confounders [68]. However, because the index incorporated several antioxidant nutrients and did not include biomarkers of oxidative stress, the findings cannot be attributed specifically to carotenoids. Nevertheless, they are consistent with broader observational evidence linking diets with greater antioxidant capacity to more favorable sleep outcomes. A systematic review similarly reported an inverse association between dietary total antioxidant capacity and sleep-related disorders [69]. In addition, higher dietary antioxidant capacity has been associated with lower odds of poor sleep among women with type 2 diabetes [70], as well as fewer self-reported sleep problems among postmenopausal women [71]. Collectively, the available evidence supports oxidative stress and endogenous antioxidant defenses as plausible pathways through which carotenoids could influence sleep; however, direct evidence demonstrating that these effects mediate improvements in sleep in humans remains limited.

### 4.4. Potential Anti-Inflammatory Mechanisms of Action of Carotenoids on Sleep

Anti-inflammatory activity may represent another mechanism through which carotenoids influence sleep regulation. Repeated sleep restriction has been associated with increased systemic and cellular inflammatory activity. In young, healthy adults, restricting sleep from 8 to 6 h per night for one week significantly increased 24 h interleukin-6 (IL-6) secretion in both sexes, while tumor necrosis factor-α (TNF-α) increased only among men [72]. Similarly, one night of partial sleep deprivation, with sleep restricted to a four-hour period from 3:00 to 7:00 a.m., significantly increased spontaneous production of IL-6 and TNF-α by peripheral blood monocytes the following morning [73]. Supporting these findings, a recent meta-analysis of 35 experimental and quasi-experimental studies reported that partial sleep deprivation (~4.3 h/night) sustained for at least three nights was associated with significant increases in circulating IL-6 and C-reactive protein (CRP) concentrations, whereas a single night of total or partial sleep deprivation did not significantly alter circulating inflammatory markers [74]. Moreover, a randomized crossover trial found that sleep restriction induces oxidative stress without upregulating antioxidant responses, suggesting that chronic insufficient sleep promotes systemic and cellular inflammatory activity without inducing compensatory clearance of endothelial oxidative stress [75].

Carotenoids may modulate inflammatory activity through inhibition of nuclear factor κB (NF-κB) signaling, a key regulator of inflammatory gene expression [25]. In lipopolysaccharide-stimulated macrophages, astaxanthin inhibited NF-κB activation, including NF-κB p65 nuclear translocation, while reducing production of the pro-inflammatory cytokines TNF-α and IL-1β [76]. Astaxanthin also inhibited IκB kinase activity and prevented degradation of IκBα, suggesting that it acts on upstream components of the NF-κB signaling pathway [76]. Additional evidence from a mouse model of reserpine-induced fibromyalgia demonstrated that astaxanthin improved sleep disturbances while reducing the expression of TNF-α, IL-6, IL-1β, and NLRP3 inflammasome components in both the anterior cingulate cortex and spinal cord [55]. Because genetic deletion of NLRP3 similarly alleviated sleep disturbances in this model, suppression of NLRP3-mediated inflammatory signaling may contribute to the sleep-promoting effects of astaxanthin [55].

Human evidence further supports the anti-inflammatory effects of carotenoids. In a 12-week RCT, supplementation with 30 mg/day of crocin significantly reduced high-sensitivity CRP, TNF-α, and NF-κB concentrations compared with placebo among adults with type 2 diabetes [77]. Similarly, supplementation with lutein, zeaxanthin, and meso-zeaxanthin for 6 months significantly reduced serum IL-1β and TNF-α concentrations [78]. Consistent with intervention findings, observational evidence found that higher serum concentrations of lutein, zeaxanthin, and total carotenoids were associated with lower odds of elevated hs-CRP [79]. Broader dietary evidence also supports a relation between inflammation and sleep, with longitudinal data demonstrating that increases in the DII are associated with greater wake after sleep onset and lower sleep efficiency [80]. Although these findings support inflammation as a plausible mechanism linking carotenoids with sleep, human studies directly evaluating changes in inflammatory markers alongside sleep outcomes are scarce, and direct evidence demonstrating that reductions in inflammation mediate improvements in sleep remains limited.

## 5. Discussion

This review contributes to a growing body of literature on the potential benefits of carotenoid intake for sleep health and is among the first to synthesize evidence from observational, clinical, and preclinical studies. Findings were largely consistent across study designs and individual studies, supporting a favorable relation between carotenoid exposure and multiple dimensions of sleep health.

Most observational studies reported beneficial associations between dietary carotenoid intake and sleep outcomes. However, substantial differences in carotenoid exposures, study populations, sleep outcomes, and sleep assessment methods limited direct comparisons across studies. Despite this methodological variability, the available evidence suggests a potential inverse U-shaped association between dietary carotenoid intake and sleep duration [27,33]. Similar trends were observed for sleep quality [28] and the presence of sleep problems [30], with higher dietary carotenoid intake generally associated with more favorable sleep outcomes. Although comparable beneficial associations were observed between serum carotenoid concentrations and sleep duration [27,36,37,38] and the presence of sleep problems [37], findings related to serum carotenoids were less consistent than those for dietary carotenoid intake [27,38]. For example, one study reported a positive association between total dietary carotenoid intake and sleep quality [27], whereas another found no significant association between serum total carotenoid concentrations and sleep quality, despite evidence that dietary carotenoid intake and circulating concentrations are highly correlated. In addition, Beydoun et al. reported that higher serum carotenoid concentrations were associated with an increased risk of short sleep after adjustment for multiple comparisons [27], in contrast with the inverse associations between serum carotenoid concentrations and short sleep reported elsewhere [36,37,38]. However, these inconsistencies may partially reflect differences between dietary intake and circulating biomarkers, such as individual genetic variation in carotenoid conversion efficiency as well as differences in intestinal carotenoid absorption capacity. These factors may also partially explain inter-individual heterogeneity in the correlation between dietary carotenoid intake and circulating carotenoid concentrations [81].

Evidence from human intervention studies similarly supports a beneficial effect of carotenoid supplementation on at least one sleep-related outcome, although the carotenoid investigated, intervention durations, dosages, and study populations differed. Nevertheless, studies using comparable interventions may provide useful guidance for future clinical trials. For example, two studies supplemented participants with doses of 10 mg/day lutein + 2 mg/day zeaxanthin using different dosing schedules, one of which reported significant improvements in subjective sleep quality with two doses daily for 4 and 8 months [41], whereas the other observed significant improvements in sleep-related impairment after 6 months of one daily dose [47]. Among studies evaluating saffron-derived apocarotenoids, supplementation with 30 mg/day of crocin for 8 weeks improved sleep quality [45], whereas no improvements were observed following 4 weeks of supplementation with 22 mg/day of saffron extract containing >3.5% Lepticrosalides^©^ [44]. In addition, both studies administering 7.5 mg/day of crocetin for 2 weeks reported significant improvements in objectively measured sleep regulation [46,50]. Unfortunately, only one identified intervention study investigated each of the more commonly consumed dietary carotenoids lycopene [42] and β-cryptoxanthin [49], with mixed findings.

All included animal studies evaluated carotenoids derived from saffron or marine algae [52,53,54,55]. Some studies suggested dose-dependent effects, with higher carotenoid doses producing greater improvements in sleep outcomes [52,53]. Animal studies further demonstrated beneficial effects on objectively measured sleep architecture, particularly non-REM sleep [53,55]. However, translation of these findings to humans may be limited because the effective doses administered in animal models substantially exceed typical human dietary intakes. Based on the corresponding conversion values [82], the effective dose of crocin, crocetin, and astaxanthin of 100 mg/kg for mice corresponds to approximately 486 mg/day for a 60 kg adult [53,55], far exceeding the average carotenoid intake of 9.7 mg/day among U.S. adults [57].

Despite the availability of a relatively large number of included studies with comparable findings across study designs, this review had several limitations. Heterogeneity in carotenoid exposure, study population, outcome measurements, and sleep assessment methods precluded a systematic analysis of the data. In addition, the cross-sectional design of the included observational studies limits assessment of temporality and precludes causal inference regarding associations between carotenoid exposure and sleep outcomes. Residual confounding and clustering of healthful lifestyle behaviors may also contribute to the observed associations, as higher carotenoid intake may occur alongside greater overall diet quality and other dietary, lifestyle, or socioeconomic characteristics that independently influence sleep. Among clinical intervention studies, variation in the carotenoid compounds, dosages, and timing of supplementation relative to other nutrient intake may have contributed to inconsistent findings and hindered identification of the optimal carotenoid type(s) and dosage for improving sleep outcomes. Inter-individual genetic differences affecting carotenoid metabolism and intestinal absorption may have also influenced responses to supplementation, limiting the generalizability of the findings and warranting further investigation. Furthermore, all human intervention studies used carotenoid supplements rather than dietary sources, limiting their applicability to free-living populations.

### Future Research Agenda

Additional research in several key areas is needed to better elucidate the role of carotenoids in multidimensional sleep health across diverse populations. Several factors that influence carotenoid bioavailability warrant further exploration, particularly diet composition and genetic variation, which may modify the association between circulating carotenoid concentrations and sleep. Unlike the intervention studies included in this review, carotenoid intake in the general population is derived primarily from fruits and vegetables rather than dietary supplements [57]. These foods contain nutrients, including fiber and unsaturated fatty acids, that independently influence both circulating carotenoid concentrations and sleep [83,84]. They also often provide other bioactive compounds, such as dietary polyphenols, that may exert independent beneficial effects on sleep [85]. Future studies should incorporate robust dietary assessment methods and appropriate statistical approaches to account for potential confounding by other dietary components when examining associations between carotenoid intake and sleep. Assessing carotenoid status using complementary biomarkers, including dietary intake, circulating serum carotenoid concentrations, and skin carotenoid concentrations measured by Raman spectroscopy, may also improve understanding of carotenoid bioavailability in relation to sleep [84,86].

Genetic variation may also contribute to inter-individual differences in carotenoid bioavailability and subsequent effects on sleep. Early research demonstrated substantial variability in plasma β-carotene responses following oral supplementation, with some individuals exhibiting marked increases and others showing only minimal responses (“nonresponders”) [81]. Subsequent preclinical research suggests that individual differences in the intestinal absorption of β-carotene and its conversion to vitamin A, as well as the metabolism of other carotenoids such as lutein and zeaxanthin, may be influenced by genetic variation within the *PKD1L2/BCO1* region [62,87]. As such, future nutrigenomic research should investigate whether genetic differences in carotenoid metabolism and intestinal absorption capacity contribute to variability in sleep responses to carotenoid intake. Furthermore, future trials should examine whether genotype modifies the sleep response to carotenoid exposure.

Future studies should prioritize dose–response investigations to determine the dose, frequency, and duration of carotenoid exposure associated with clinically meaningful changes across multiple dimensions of sleep health and whether these relationships differ by carotenoid type. To better inform clinical translation, these relations should be examined using carotenoids derived from both dietary supplements and whole foods and across diverse populations. Greater consistency in sleep assessment is also needed, including standardized sleep outcomes, measurement methods, and definitions of key sleep metrics, such as sleep quality and duration. Future studies should employ validated subjective and objective sleep assessment methods, such as the PSQI and actigraphy, respectively, and adopt recognized definitions of key sleep metrics, including short and long sleep durations, where available [1,88]. Greater standardization would improve comparability across studies, facilitate future quantitative synthesis, and strengthen the evidence needed to inform clinical recommendations.

Further investigation is also warranted into the sociodemographic, cultural, and health-related factors that may influence associations between carotenoid intakes and sleep. Carotenoid intake varies according to demographic characteristics [57], geographic location [89], and socioeconomic status [90], reflecting differences in food cultures, food environments, and overall diet quality. Similar cross-cultural variation is observed in sleep patterns [91,92], underscoring the need for culturally informed studies examining the relation between carotenoid intake and sleep across diverse populations. In addition, research is needed to determine whether carotenoids uniquely influence sleep among individuals with pro-inflammatory cardiometabolic conditions, including cardiovascular disease and type 2 diabetes, in whom sleep health plays an important role in disease progression [1,7]. Such work would clarify whether sleep mediates associations between carotenoid intake and chronic disease while improving generalizability and informing equitable dietary and lifestyle recommendations aimed at reducing chronic disease risk.

## 6. Conclusions

Emerging evidence suggests that greater carotenoid intake may be associated with more favorable outcomes across multiple dimensions of sleep health, with antioxidant and anti-inflammatory pathways providing biologically plausible mechanisms. However, direct evidence from human intervention studies remains limited and is insufficient to recommend specific carotenoids, doses, or supplementation strategies for improving sleep. Future research should prioritize well-designed dose–response human intervention trials evaluating individual carotenoids from both whole foods and dietary supplements, with standardized sleep outcomes and sufficient population diversity to identify potential differences in response. Carotenoid-rich foods may continue to be encouraged within existing healthful diet recommendations but should not be specifically prescribed as a strategy to improve sleep until efficacy is established.

## Figures and Tables

**Table 1:** Summary of human observational studies exploring the association between carotenoid intake and sleep parameters.

Reference	Study Design	Sample Size (*n*), Population	% Female (*n*); Age (Mean ± SD or Range)	Primary Exposure(s)	Primary Sleep Outcome(s); Measurement Method	Main Findings
Beydoun et al., 2014 [27]	Cross-sectional	*n* = 2459 Adults from NHANES 2005–2006	Undeclared for the analytical sample; however, 52.1% (*n* = 2592) of the total sample (*n* = 4979) were female 20–85 years	Serum concentrations and dietary intake of total carotenoids, α-carotene, β-carotene, β-cryptoxanthin, lutein + zeaxanthin, lycopene	Sleep duration, presence of sleep disorders, sleep quality, daytime dysfunction, sleepiness, sleep disturbance; Functional Outcomes of Sleep Questionnaire	Compared to those with normal sleep duration (7–8 h), those with short (5–6 h) and very short (<5 h) sleep duration had significantly lower **serum levels** of α-carotene, β-carotene, total carotenoids, and β-cryptoxanthin (short sleep duration only). Compared to those with normal sleep duration, those with short and very short sleep duration had significantly lower **dietary intakes** of α-carotene, β-carotene, lutein + zeaxanthin, and total carotenoids. Compared to those with normal sleep duration, those with long sleep duration (≥9 h) had significantly lower **dietary intakes** of lutein + zeaxanthin and total carotenoids. *In models controlling for multiple testing:* - Higher serum α-carotene: decreased sleepiness; - Higher serum β-carotene, β-cryptoxanthin: greater sleepiness; - Higher serum lutein + zeaxanthin: greater daytime dysfunction, lower sleep disturbance; - Higher serum total carotenoids: greater sleepiness, lower sleep disturbance; - Total serum carotenoids were associated with 15% higher risk of short sleep.
Boozari et al., 2022 [28]	Cross-sectional	*n* = 368 Healthy university students	51% (*n* = 187) female 22.9 ± 3.9 years	Dietary total carotenoid intake	Sleep quality; PSQI	Those with higher carotenoid intake were less likely to have poor sleep quality. Compared with Q1 (0.06–3.85 mg/day), participants in Q3 (7.25–12.17 mg/day) and Q4 (12.18–18.28 mg/day) of carotenoid intake had 71% and 81% lower adjusted odds of poor sleep quality (PSQI score ≥ 6), respectively. Associations remained significant in sex-stratified analyses.
Çakir et al., 2020 [29]	Cross-sectional	*n* = 2446 Adults aged 20–64 years	51.1% (*n* = 1250) female 38.7 ± 12.7 years	Dietary β-carotene intake	Sleep duration, sleep quality; PSQI	Those with good sleep quality (PSQI score ≤ 5) had significantly higher daily dietary β-carotene intake than those with poor sleep quality (PSQI score > 5). There were no significant differences in β-carotene intake across short (≤6 h), normal (>6–≤8 h), and long (>8 h) sleep-duration categories.
Chen et al., 2023 [30]	Cross-sectional	*n* = 24,234 Adults from NHANES 2005–2018	46.4% (*n* = 11,251) female ≥18 years; 68.3% < 60 years	Dietary carotenoid intake (α-carotene, β-carotene, β-cryptoxanthin, lutein + zeaxanthin, lycopene)	Presence of sleep problems; Sleep Habits and Disorders Questionnaire	Compared with Q1, participants in Q3 of lycopene intake had 8.1% lower adjusted odds of sleep problems; however, no significant overall trend across quartiles was observed. No significant overall associations were observed for the other carotenoids examined. *These quartiles had a significantly lower prevalence of sleep problems compared to intake Q1 when stratified by sociodemographic factors:* - Those in Q4 of α-carotene intake who were 60 years of age or older, had a BMI > 30 kg/m^2^, or had 1–2 physical activity episodes/week. - Those in Q2 of β-carotene intake who were female or did not drink alcohol. - Those in Q4 of β-carotene intake who had a BMI > 30 kg/m^2^ or had 1–2 physical activity episodes/week. - Those in Q2 of β-cryptoxanthin intake who did not drink alcohol or had 0 physical activity episodes/week. - Those in Q3 of lycopene intake who were female, less than 60 years of age, had a BMI 25–30 kg/m^2^, or were smokers. - Those in Q4 of lycopene intake who were female or had 0 physical activity episodes/week.
Day et al., 2009 [31]	Cross-sectional case–control	*n* = 18 (9 OSA patients, 9 age- and sex-matched controls) Adults with OSA and controls without clinical suspicion of OSA	11.1% (*n* = 2) female OSA group: 43.7 ± 2.9 years Control group: 44.3 ± 3.5 years	Pre-sleep and post-sleep serum carotenoid concentrations (β-carotene [all-trans, 13-cis, 9-cis], β-cryptoxanthin, lutein, zeaxanthin, lycopene)	OSA (compared to matched healthy controls)	Pre-sleep plasma concentrations of all-trans and 9-cis β-carotene were significantly lower in OSA patients compared with controls (50% and 35% lower, respectively). No significant differences were observed for other carotenoids or in carotenoid concentrations before vs. after sleep in either group.
Degenhard et al., 2025 [32]	Cross-sectional	*n* = 6730 Adults from NHANES 2011–2014	52% (*n* = 3500) female 47.5 ± 0.45 years	Dietary carotenoid intake (β-carotene and lycopene)	Sleep regularity index; Actigraphy watch	Higher dietary β-carotene and lycopene intakes were positively associated with the sleep regularity index score.
Deng et al., 2023 [33]	Cross-sectional	*n* = 23,307 Adults from NHANES 2007–2018	52.2% (*n* = 12,087) female Average age: 47.5 years	Dietary carotenoid intake (total carotenoids, α-carotene, β-carotene, β-cryptoxanthin, lutein + zeaxanthin, lycopene)	Sleep duration; Home-based questionnaire	Those with optimal sleep duration (7–8 h) had higher intakes of all carotenoids (α-carotene, β-carotene, β-cryptoxanthin, lutein + zeaxanthin, lycopene) compared to short (<7 h) or long (>8 h) sleepers. Compared with optimal sleep duration, those with higher dietary intakes of α-carotene, β-carotene, β-cryptoxanthin, lutein + zeaxanthin and lycopene were significantly less likely to be short sleepers. Compared with optimal sleep duration, long sleepers consumed significantly less α-carotene, β-carotene, β-cryptoxanthin, lutein + zeaxanthin and lycopene. A dose–response association suggests a reverse U-shaped relationship between sleep duration and dietary carotenoid intake. Combined carotenoid intake significantly reduced the odds of short and long sleep duration. β-cryptoxanthin and lutein + zeaxanthin had the strongest association with lower odds of short sleep duration, while α-carotene, β-carotene, and β-cryptoxanthin had the strongest association with lower odds of long sleep duration.
Grandner et al., 2014 [34]	Cross-sectional	*n* = 4552 Adults from NHANES 2007–2008	53.1% (*n* = 2417) female 46.3 ± 16.5 years	Dietary carotenoid intake (α-carotene, β-carotene, β-cryptoxanthin, lutein + zeaxanthin, lycopene)	Sleep symptoms (difficulty falling asleep, difficulty maintaining sleep, non-restorative sleep, daytime sleepiness); Questions as part of a larger survey	A 100% higher α-carotene intake was significantly associated with 4% lower odds of having difficulty falling asleep. A 100% higher lycopene intake was significantly associated with 2% lower odds of having difficulty maintaining sleep.
Grandner et al., 2013 [35]	Cross-sectional	*n* = 4548 Adults from NHANES 2007–2008	53.1% (*n* = 2415) female 46.3 ± 16.5 years	Dietary carotenoid intake (α-carotene, β-carotene, β-cryptoxanthin, lutein + zeaxanthin, lycopene)	Sleep duration; Question as part of a larger survey	Compared to normal sleep duration (7–8 h), very short sleep duration (<5 h) was associated with lower dietary lycopene intake. Short sleep (5–6 h) was associated with higher lutein + zeaxanthin intake.
Kanagasabai et al., 2015 [36]	Cross-sectional	*n* = 2079 Adults from NHANES 2005–2006	50.7% (*n* = 1054) female Average age: 46.0–49.5 years	Serum carotenoid concentration	Sleep duration; Sleep Disorders Questionnaire	Compared to 7–8 h sleep duration, short (5–6 h) or very short (≤4 h) sleep duration was associated with significantly lower serum carotenoid levels.
Kanagasabai & Ardern, 2015 [40]	Cross-sectional	*n* = 2072 U.S. adults from NHANES 2005–2006	~49% (*n* = 1015) female Aged ≥ 20 years	Serum carotenoid concentration	Sleep quality; Sleep Disorders Questionnaire	Serum carotenoid concentration did not differ significantly across sleep quality categories.
Tang et al., 2024 [37]	Cross-sectional	*n* = 3062 U.S. adults from NHANES 2017–2018	49.1% (*n* = 1504) female Aged ≥ 20 years	Serum carotenoid concentrations (α-carotene, α-cryptoxanthin, trans-β-carotene, β-cryptoxanthin, lutein + zeaxanthin, trans-lycopene, and total lycopene)	Trouble sleeping, sleep duration, frequent snoring, and snorting or stopping breathing; Sleep Habits and Disorders Questionnaire	Higher serum α-carotene, α-cryptoxanthin, trans-β-carotene, β-cryptoxanthin, and lutein + zeaxanthin were significantly associated with lower odds of overall trouble sleeping and abnormal sleep duration. Higher serum α-carotene, α-cryptoxanthin, trans-β-carotene, and lutein + zeaxanthin were associated with lower odds of frequent snoring. Higher serum α-cryptoxanthin, trans-β-carotene, and lutein + zeaxanthin concentrations were associated with lower odds of frequent snorting or stopping breathing.
Matsunaga et al., 2021 [39]	Cross-sectional	*n* = 124 Japanese male university students	0% (*n* = 0) female 21.4 ± 2.4 years	Dietary β-carotene equivalent intake	Sleep quality; Japanese PSQI	After multivariable adjustment, participants with poor sleep quality (PSQI ≥6) had significantly lower β-carotene equivalent intake than those with good sleep quality (PSQI ≤ 3). Higher PSQI scores (worse sleep quality) were inversely associated with β-carotene intake.
Noorwali et al., 2018 [38]	Cross-sectional	*n* = 1612 U.K. adults from the National Diet and Nutrition Survey 2008/2009–2011/2012	57% (*n* = 919) female 43.1 (95% CI: 43–44) years	Plasma total carotenoids, α-carotene, β-carotene, and lycopene	Weighted average of self-reported weekday and weekend sleep duration	Compared with adults sleeping 7–8 h/night, short sleepers had significantly lower plasma total carotenoids, β-carotene, and lycopene. Plasma α-carotene did not differ significantly.

Abbreviations: BMI, body mass index; NHANES, National Health and Nutrition Examination Survey; OSA, obstructive sleep apnea; PSQI, Pittsburgh Sleep Quality Index; SD, standard deviation; U.K., United Kingdom.

**Table 2:** Summary of human intervention studies exploring the association between carotenoid intake and sleep parameters.

Reference	Carotenoid Class	Carotenoid Compound	Study Design	Sample Size (*n*), Population	% Female (*n*), Age (Mean ± SD or Range)	Dose, Days, Timing	Comparator	Primary Sleep Outcome(s), Measurement Method	Main Findings
Bharadwaj et al., 2025 [41]	Xanthophyll	Lutein + zeaxanthin	Crossover RCT	*n* = 60 (*n* = 20 per group) Healthy adults with ≥8 h/day of screen exposure	53.3% (*n* = 32) female 37.1 ± 11.9 years	10 mg of lutein + 2 mg of zeaxanthin twice daily Group A: 4 months of intervention + 15-day washout + 4 months of intervention Group B: 4 months of intervention + 15-day washout + 4 months of placebo Group C: 4 months of placebo + 15-day washout + 4 months of intervention Timing: NR	Placebo	Sleep quality metrics (daily hours of sleep, sleep frequency, wake frequency, feelings of relaxation after sleep, feeling of eyes after sleep) Author-developed Quality of Sleep Questionnaire	Across all three groups, most sleep quality metrics significantly improved during the study visits (midpoint or endpoint vs. baseline) after the treatment with lutein + zeaxanthin. In groups B and C, results were largely non-significant after the placebo was compared to baseline or end of treatment with lutein + zeaxanthin.
Carrasco et al., 2022 [42]	Carotene	Lycopene	Pilot study	*n* = 20 Group A: healthy men (*n* = 10) Group B: men with benign prostatic hyperplasia (*n* = 10)	0% (*n* = 0) female ≥50 years	8 mg of lycopene daily for 30 days via enriched extra-virgin olive oil Timing: At breakfast and/or lunch	N/A	Sleep quality Wrist Actigraphy	In both groups, the percentage of night activity significantly decreased postintervention compared with baseline. No significant changes were observed in minutes of nighttime sleep or depth of sleep in either group.
Hayashi et al., 2020 [43]	Xanthophyll	Astaxanthin	RCT	*n* = 54 (placebo group = 29; active group = 25) Healthy adults with elevated Depression–Dejection scores	Placebo group: 58.6% (*n* = 17) female; 45.3 ± 9.3 years Active group: 56.0% (*n* = 14) female; 45.8 ± 10.5 years	2 mg of astaxanthin 6×/day (12 mg/day total) for 8 weeks Timing: ½ after breakfast and ½ after dinner	Placebo	Sleepiness on rising; initiation and maintenance of sleep; frequent dreaming; refreshing; sleep length OSA-MA questionnaire	After 4 weeks of astaxanthin supplementation, sleepiness on rising and refreshing significantly improved from baseline in the active group. After 8 weeks of supplementation, sleepiness on rising significantly improved from baseline in the active group. Although there were no significant between-group differences in sleep outcomes, sleepiness on rising significantly improved with astaxanthin compared to placebo among a subgroup of participants with higher baseline Depression–Dejection scores (>65).
Kell et al., 2017 [44]	Apocarotenoid ^a^	Saffron extract (crocin + safranal)	RCT	*n* = 121 (group A = 41; group B = 42; placebo group = 38) Healthy adults with self-reported low mood	62.0% (*n* = 75) female 39.1 ± 13.8 years	Group A: 28 mg/day saffron extract (>3.5% Lepticrosalides^®^) ^b^ Group B: 22 mg/day saffron extract (>3.5% Lepticrosalides^®^) ^b^ 4 weeks Timing: ½ at morning meal and ½ at midday meal	Placebo	Sleep quality PSQI	There were no significant improvements in sleep quality in any of the treatment groups.
Khalatbari-Mohseni et al., 2019 [45]	Apocarotenoid ^a^	Crocin	RCT	*n* = 50 (25 per group) Adult males with a confirmed diagnosis of substance dependency on methadone maintenance treatment	0% (*n* = 0) female Intervention group: 40.1 ± 9.3 years Placebo group: 41.4 ± 8.8 years	15 mg crocin 2×/day (30 mg/day total) for 8 weeks Timing: one hour after food	Placebo	Sleep quality PSQI	Compared with placebo, 8 weeks of crocin supplementation significantly improved sleep quality.
Kuratsune et al., 2010 [46]	Apocarotenoid ^a^	Crocetin	Pilot crossover RCT	*n* = 21 Healthy adult males with poor sleep quality (PSQI score ≥6)	0% (*n* = 0) female 43.9 ± 8.5 years	7.5 mg crocetin daily for 2 weeks + 2-week washout period + 2 weeks placebo Timing: between 6:00 pm and 8:00 pm	Placebo	Objective (Actigraphy watch): activity mean, sleep latency, sleep efficiency, wake episodes Subjective (St Mary’s Hospital Sleep Questionnaire): sleep quality	The number of wake episodes was significantly lower in the crocetin group compared to the placebo group at the end of the intervention. Subjective sleep quality showed non-significant trends towards improvement with crocetin.
Lopresti et al., 2025 [47]	Xanthophyll	Lutein + zeaxanthin	RCT	*n* = 60 (intervention group = 29; placebo group = 31) Healthy adults with ≥6 h/day of screen time	Undeclared for the analytical sample; full sample (*n* = 70) detailed. Intervention group: 45.7% (*n* = 16) female; 48.3 ± 12.1 years Placebo group: 45.7% (*n* = 16) female; 46.1 ± 12.0 years	10 mg lutein + 2 mg zeaxanthin isomers daily for 180 days (6 months) Timing: with a meal	Placebo	Sleep disturbance, sleep-related impairment PROMIS Sleep Disturbance and Sleep-Related Impairment Scale	There were no significant between-group differences in either sleep disturbance or sleep-related impairment scores. Sleep-related impairment significantly improved from baseline to endpoint in both the intervention and placebo groups.
Stringham et al., 2017 [48]	Xanthophyll	Lutein + zeaxanthin + meso-zeaxanthin	RCT	*n* = 48 (intervention group = 35; placebo group = 13) Healthy college-aged adults with ≥6 h/day of near-field screen exposure	52.1% (*n* = 25) female Average age 21.2 years	24 mg/day macular carotenoids for 6 months (lutein, zeaxanthin, and meso-zeaxanthin in an 83%:10%:7% ratio) Timing: with a meal (preferably lunch or dinner)	Placebo	Sleep quality PSQI	Compared with placebo, macular carotenoid supplementation significantly improved sleep quality after 3 and 6 months, with a stronger effect observed at 6 months. The mean number of undesirable sleep symptoms decreased from 4.74 at baseline to 3.89 at 3 months and 3.80 at 6 months in the intervention group.
Tan et al., 2023 [49]	Xanthophyll	β-cryptoxanthin	RCT	*n* = 80 (group A = 26; group B = 26; placebo group = 28) Healthy young adult women	100% (*n* = 80) female Group A: 22.5 ± 7.8 years Group B: 25.5 ± 7.5 years Placebo group: 27.5 ± 8.5 years	Group A: 3 mg/day β-cryptoxanthin Group B: 6 mg/day β-cryptoxanthin 8 weeks Timing: NR	Placebo	Subjective (PSQI): sleep quality Objective (wrist actigraphy): sleep duration	There were no significant within-group or between-group changes in subjective sleep quality or objectively measured sleep duration.
Umigai et al., 2018 [50]	Apocarotenoid ^a^	Crocetin	Crossover RCT	*n* = 24 Healthy men and postmenopausal women with mild sleep complaints	41.7% (*n* = 10) female 50.8 ± 6.9 years	7.5 mg/day crocetin for 14 days + 14-day washout + 14 days placebo Timing: after evening meal	Placebo	Subjective (OSA-MA): sleepiness on rising; initiation and maintenance of sleep; frequent dreaming; feeling refreshed; sleep length Objective (EEG): delta power, sleep latency, REM sleep latency, sleep efficiency, WASO, total sleep time	EEG-derived delta power was significantly higher with crocetin intake compared to placebo. There were significant improvements in sleepiness on rising and feeling refreshed after crocetin supplementation compared to placebo.
Yoo et al., 2024 [51]	Xanthophyll	Fucoxanthin	RCT	*n* = 43 (intervention group = 22; placebo group = 21) Healthy adults aged 55–75 years with age-associated memory impairment	46.5% (*n* = 20) female 64.3 ± 6.0 years	1100 mg/day *Phaeodactylum tricornutum* extract containing 8.8 mg fucoxanthin for 12 weeks Timing: at lunchtime	Placebo	Sleep quality: ease of going to sleep; awakening from sleep; perceived quality of sleep; integrity of behavior following wakefulness Leeds Sleep Evaluation Questionnaire	At 4 weeks, the fucoxanthin group showed significant improvements from baseline in awakening and time to feeling alert after waking, while calmer sleep showed a non-significant trend. No significant overall group × time effect was observed.

^a^ Derived from the carotenoid zeaxanthin, a xanthophyll-class carotenoid. ^b^ Measure of bioactive compounds in saffron that includes crocin and safranal. Abbreviations: EEG, electroencephalogram; NR, not reported; OSA-MA, Oguri–Shirakawa–Azumi Sleep Inventory, Middle-Aged and Aged version; PROMIS, Patient-Reported Outcomes Measurement Information System; PSQI, Pittsburgh Sleep Quality Index; REM, rapid eye movement; RCT, randomized controlled trial; SD, standard deviation; WASO, wake after sleep onset.

**Table 3:** Summary of animal studies exploring the association between carotenoid intake and sleep parameters.

Reference	Carotenoid Class	Carotenoid Compound	Dose	Control	Animal Model	Main Findings
Hosseinzadeh et al., 2009 [52]	Apocarotenoid ^a^	Crocin, safranal	Crocin: 50, 200, and 600 mg/kg IP Safranal: 0.05, 0.15, and 0.35 mL/kg IP	10 mL/kg saline (crocin vehicle) Paraffin (safranal vehicle) 3 mg/kg diazepam (reference)	Razi male mice	Safranal dose-dependently increased pentobarbital-induced sleeping time, whereas crocin had no significant effect.
Masaki et al., 2012 [53]	Apocarotenoid ^a^	Crocin, crocetin	Crocin mice: 10, 30, or 100 mg/kg IP Crocetin mice: 100 mg/kg IP	Vehicle control	Male C57BL/6 strain mice	Crocin at 30 and 100 mg/kg increased non-REM sleep by 60% and 170% and decreased wakefulness by 20% and 50%, respectively, without affecting REM sleep; 10 mg/kg had no significant effect. At 100 mg/kg, crocin also increased non-REM and wake bouts, shortened wake episodes, and increased transitions between wakefulness and non-REM sleep. Crocetin at 100 mg/kg increased non-REM sleep by 60% and decreased wakefulness by 25%, without affecting REM sleep.
Moskalev et al., 2018 [54]	Xanthophyll	Fucoxanthin	1 μM fucoxanthin in yeast paste, administered beginning on the first day of adult life	Yeast paste without fucoxanthin	Male and female wild-type Canton-S *Drosophila melanogaster*	Fucoxanthin increased nocturnal sleep/rest (defined as ≥5 consecutive minutes of inactivity) in males of all ages and in 6-week-old females. Daytime sleep/rest varied by age and sex, with reduced sleep in 1-week-old males and females and increased sleep in 4-week-old males and 6-week-old females.
Zhao et al., 2024 [55]	Xanthophyll	Astaxanthin	AST1: 10 mg/kg/day administered intragastrically for 10 days AST2: 50 mg/kg/day administered intragastrically for 10 days AST3: 100 mg/kg/day administered intragastrically for 10 days *	Fibromyalgia control mice	Female C57BL/6 mice with reserpine-induced fibromyalgia	At 100 mg/kg/day, astaxanthin significantly decreased wake duration and the number of wake bouts and significantly increased non-REM sleep duration compared with fibromyalgia control mice. No significant change in REM sleep duration was observed.

* Sleep outcomes were only reported for AST3. ^a^ Derived from the carotenoid zeaxanthin, a xanthophyll-class carotenoid. Abbreviations: IP, intraperitoneal; REM, rapid eye movement.

## Data Availability

No new data were created or analyzed in this study. Data sharing is not applicable to this article.

## Supplementary Materials

The following supporting information can be downloaded at: https://www.mdpi.com/article/10.3390/nu18172833/s1. Search Strategy.
