# Supplementary material for: Dietary Carotenoid Intake and Sleep: A Narrative Review of Evidence from Human and In Vivo Animal Studies
Source: Nutrients. 2026 Aug 28;18(17):2833. doi: 10.3390/nu18172833 (PMC13566979; doi:10.3390/nu18172833)
Supplement: Supplementary file 1 [file nutrients-18-02833-s001.zip › nutrients-4495798-Supplementary.pdf]

Dietary Carotenoid Intake and Sleep: A Narrative Review of Evidence from Human and In Vivo Animal Studies

Supplementary Material: Search Strategy

PubMed: 879 results (after Pubmed filter for 'Language = English'; no duplicates)

("Carotenoids"[Mesh] OR carotenoid*[tiab] OR "dietary carotenoid*"[tiab] OR "carotenoid intake"[tiab] OR "carotenoid consumption"[tiab] OR "dietary intake"[tiab] OR "alpha-carotene"[tiab] OR α-carotene[tiab] OR "beta-carotene"[Mesh] OR "beta-carotene"[tiab] OR β-carotene[tiab] OR "beta-cryptoxanthin"[tiab] OR β-cryptoxanthin[tiab] OR lycopene[Mesh] OR lycopene[tiab] OR lutein[Mesh] OR lutein[tiab] OR zeaxanthin[Mesh] OR zeaxanthin[tiab] OR astaxanthin[Mesh] OR astaxanthin[tiab] OR ((carotenoid* OR "alpha-carotene" OR "beta-carotene" OR "beta-cryptoxanthin" OR lycopene OR lutein OR zeaxanthin OR astaxanthin) AND (supplement* OR "dietary supplement*" OR nutraceutical* OR capsule* OR fortified))) AND ("Sleep"[Mesh] OR "Sleep Wake Disorders"[Mesh] OR "Sleep Initiation and Maintenance Disorders"[Mesh] OR "Sleep Apnea Syndromes"[Mesh] OR sleep[tiab] OR "sleep quality"[tiab] OR "sleep duration"[tiab] OR insomnia[tiab] OR "sleep disorder*"[tiab] OR "sleep-disordered breathing"[tiab] OR "sleep apnea"[tiab] OR OSA[tiab] OR "obstructive sleep apnea"[tiab] OR "sleep architecture"[tiab] OR "REM sleep"[tiab] OR "rapid eye movement"[tiab] OR "slow wave sleep"[tiab] OR "deep sleep"[tiab] OR "light sleep"[tiab]) NOT (review[pt] OR systematic review[pt] OR meta-analysis[pt] OR editorial[pt] OR comment[pt] OR letter[pt])

Scopus: 262 results (after Scopus filter for 'Language = English; Document Type = Article'; 99 duplicates)

(TITLE-ABS-KEY (carotenoid* OR "alpha-carotene" OR α-carotene OR "beta-carotene" OR β-carotene OR "beta-cryptoxanthin" OR β-cryptoxanthin OR lycopene OR lutein OR zeaxanthin OR astaxanthin) OR TITLE-ABS-KEY ((carotenoid* OR "alpha-carotene" OR "beta-carotene" OR "beta-cryptoxanthin" OR lycopene OR lutein OR zeaxanthin OR astaxanthin) W/3 (supplement* OR "dietary supplement*" OR nutraceutical* OR capsule* OR fortified))) AND TITLE-ABS-KEY (sleep OR "sleep quality" OR "sleep duration" OR insomnia OR "sleep disorder*" OR "sleep-disordered breathing" OR "sleep apnea" OR "obstructive sleep apnea" OR OSA OR "sleep architecture" OR "REM sleep" OR "rapid eye movement" OR "slow wave sleep" OR "deep sleep" OR "light sleep") AND NOT DOCTYPE (re) AND NOT DOCTYPE (ed) AND NOT DOCTYPE (le) AND (LIMIT-TO (DOCTYPE , "ar")) AND (LIMIT-TO (LANGUAGE , "English"))

Hand Search: 1 result (1 duplicate)
